# Physiological Plasticity Is Important for Maintaining Sugarcane Growth under Water Deficit

**DOI:** 10.3389/fpls.2017.02148

**Published:** 2017-12-20

**Authors:** Paulo E. R. Marchiori, Eduardo C. Machado, Cristina R. G. Sales, Erick Espinoza-Núñez, José R. Magalhães Filho, Gustavo M. Souza, Regina C. M. Pires, Rafael V. Ribeiro

**Affiliations:** ^1^Department of Biology, Federal University of Lavras, Lavras, Brazil; ^2^Laboratory of Plant Physiology ‘Coaracy M. Franco’, Center for Research and Development in Ecophysiology and Biophysics, Agronomic Institute (IAC), Campinas, Brazil; ^3^Lancaster Environment Centre, Lancaster University, Lancaster, United Kingdom; ^4^Laboratory of Plant Physiology and Biotechnology, Department of Engineering and Agricultural Sciences, National University Toribio Rodríguez de Mendoza de Amazonas (UNTRM), Chachapoyas, Peru; ^5^Department of Botany, Institute of Biology, Federal University of Pelotas, Capão do Leão, Brazil; ^6^Section of Irrigation and Drainage, Center for Research and Development in Ecophysiology and Biophysics, Agronomic Institute (IAC), Campinas, Brazil; ^7^Department of Plant Biology, Institute of Biology, University of Campinas, Campinas, Brazil

**Keywords:** phenotypic plasticity, drought tolerance, root growth, *Saccharum* spp.

## Abstract

The water availability at early phenological stages is critical for crop establishment and sugarcane varieties show differential performance under drought. Herein, we evaluated the relative importance of morphological and physiological plasticity of young sugarcane plants grown under water deficit, testing the hypothesis that high phenotypic plasticity is associated with drought tolerance. IACSP95-5000 is a high yielding genotype and IACSP94-2094 has good performance under water limiting environments. Plants were grown in rhizotrons for 35 days under three water availabilities: high (soil water matric potential [Ψ_m_] higher than -20 kPa); intermediate (Ψ_m_ reached -65 and -90 kPa at the end of experimental period) and low (Ψ_m_ reached values lower than -150 kPa). Our data revealed that morphological and physiological responses of sugarcane to drought are dependent on genotype and intensity of water deficit. In general, IACSP95-5000 showed higher physiological plasticity given by leaf gas exchange and photochemical traits, whereas IACSP94-2094 showed higher morphological plasticity determined by changes in leaf area (LA) and specific LA. As IACSP94-2094 accumulated less biomass than IACSP95-5000 under varying water availability, it is suggested that high morphological plasticity does not always represent an effective advantage to maintain plant growth under water deficit. In addition, our results revealed that sugarcane varieties face water deficit using distinct strategies based on physiological or morphological changes. When the effectiveness of those changes in maintaining plant growth under low water availability is taken into account, our results indicate that the physiological plasticity is more important than the morphological one in young sugarcane plants.

## Introduction

The global interest in sugarcane (*Saccharum* spp.) production has increased significantly in the last years due to its importance as a source of renewable energy. As a semi-perennial species, sugarcane plants are commonly exposed to sub-optimal or limiting environmental conditions during growing season, which affects crop yield and biomass production. Among the environmental constrains, drought is the main abiotic factor causing yield losses in sugarcane (Basnayake et al., 2012), especially when it occurs during the initial developmental stages (Machado et al., 2009).

Under drought, tissue dehydration may reach critical levels and interfere in cellular homeostasis (Miller et al., 2010), increasing cell damage (Filippou et al., 2011) and changing plant metabolism and growth. Drought-induced limitations on sugarcane photosynthetic performance are caused by low CO_2_ diffusion to carboxylation sites associated with impairments on biochemical and photochemical reactions, i.e., physiological responses (Ghannoum, 2009; Ribeiro et al., 2013; Sales et al., 2013; Zhang et al., 2015). Sugarcane plants also show important morphological responses under water deficit, such as reduction of leaf area (LA) for preventing water loss and further dehydration (Lopes et al., 2011). Changes in root system are also reported under water deficit (Smith et al., 2005), with such responses being genotype-dependent (Jangpromma et al., 2012; Sales et al., 2012).

Several studies on sugarcane responses to drought revealed high genotypic variation among sugarcane varieties under low water availability (Basnayake et al., 2012; Jangpromma et al., 2012; Ribeiro et al., 2013; Sales et al., 2013; Khueychai et al., 2015). However, the relative importance of morphological and physiological processes underlying drought acclimation is still poorly understood, even being an important issue for crop yield and high resource use efficiency (Parry and Hawkesford, 2010). In fact, the effectiveness of morphological and physiological responses of sugarcane for maintaining growth under water deficit is unknown as most of studies evaluated only physiological changes or only morphological ones. Another relevant aspect is that most of studies were performed in well-established plants and we know that sugarcane yield is severely reduced when water stress occurs at early developmental stages (Ramesh, 2000; Machado et al., 2009).

Given the strong connection between plants and their surrounding environment, the morpho-physiological responses are dependent on the plants’ ability to perceive and react to the environmental change. This ability to express alternative phenotypes in a changing environment is known as phenotypic plasticity (Valladares et al., 2006) and it is suggested that the plasticity of some functional traits can benefit plant growth in adverse environmental conditions (Matesanz et al., 2010). Under low water availability, drought tolerant species would be recognized by their ability in reducing plant metabolism and presenting morpho-physiological changes in order to overcome the stressful condition (Souza and Luttge, 2015).

In this context, we tested the hypothesis that high phenotypic plasticity is associated with drought tolerance in sugarcane plants. Besides, our aim was to evaluate the relative importance of morphological and physiological plasticity in sugarcane plants under varying water availability at the initial developmental phase. Two sugarcane genotypes with differential stalk yield were evaluated herein and the concepts related to drought tolerance, plant performance and phenotypic plasticity discussed.

## Materials and Methods

### Plant Material and Growth Conditions

Sugarcane plants (*Saccharum* spp.) were grown under greenhouse conditions, where average air temperature was 23.8 ± 2.7°C, the mean air vapor pressure deficit (VPD) was 2.71 ± 1.03 kPa and the maximum photosynthetic active radiation (*Q*) was around 1,100 μmol m^-2^ s^-1^. We evaluated two sugarcane varieties developed by the Sugarcane Breeding Program of the Agronomic Institute (ProCana, IAC, Brazil). The variety IACSP95-5000 is responsive to agricultural inputs, showing high cane yield under favorable conditions, whereas IACSP94-2094 is less responsive to agricultural inputs, with reasonable yield in marginal areas (Landell et al., 2005, 2007) due to its drought tolerance (Ribeiro et al., 2013).

Mini-stalks (around 3 cm length) with one bud were planted in soilless substrate composed by sphagnum peat and expanded vermiculite (Carolina Soil^®^, Brazil). Twenty days after sprouting, the plants were transplanted to rhizotrons (0.95 m depth × 0.26 m diameter) filled with soil (59% coarse sand; 13% fine sand; 22% silt and 6% clay). At this time, plant height (until the first leaf insertion) was around 6 cm, showing none or one totally expanded leaf, the sett roots were present and their length varied between 5 and 10 cm. Just after transplantation, all plants were well-watered (0.3 L) for the establishment of root system in the new environment.

Rhizotrons were built with a fixed window glass and a movable black acrylic cover in order to keep roots under dark. They were positioned at 45° angle with the horizontal plane to force root growth along the window glass. Three driplines with one self compensated dripper (Netafim, Israel) each were installed in each rhizotron, totaling three drippers per rhizotron at the depths of 0.15, 0.45, and 0.75 m from soil surface. Each dripper had a flow rate of 2 L h^-1^. Granular soil matrix sensors (200SS, WaterMark^®^, Irrometer, United States) were installed at the same depths of drippers to monitor soil water, totaling three sensors in each rhizotron. There were water flow controllers in each emitter to enable independent water supplying and maintain the same water availability along the rhizotron profile. The irrigation system was composed by two main lines that carried water to secondary lines installed inside the rhizotrons. The irrigation system was driven by 0.5 hp electric pump (Hydrobloc P500, KSB, Brazil).

**FIGURE 1:**
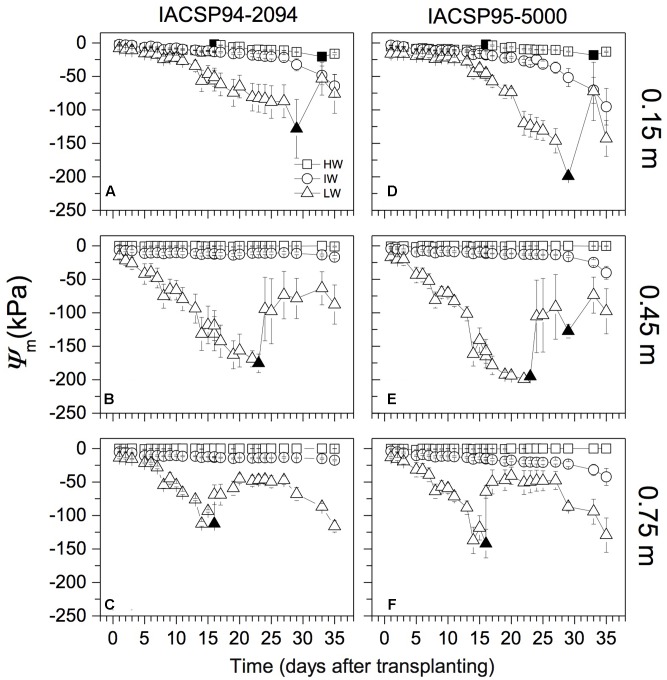
Water matric potential (Ψ_m_) along the soil profile under three water regimes: high (HW), intermediate (IW), and low (LW) water availability. Two sugarcane varieties were grown (IACSP94-2094 in **A–C**, and IACSP95-5000 in **D–F**) and each symbol is the mean value (±SD) of three replications, with exception of data for LW at **(C)** (*n* = 2). Closed symbols indicate irrigation.

Sugarcane plants were grown under three water regimes based on soil water matric potential (Ψ_m_): high (HW); intermediate (IW); and low (LW) water availability. At the beginning of the experiment, rizotrons received as initial water volume 4.4, 2.2, and 1.3 L in HW, IW, and LW treatments, respectively. After this time, the water management was done for maintaining Ψ_m_ higher than -20, -90, and -150 kPa through soil profile in HW, IW, and LW treatments, respectively. As consequence, two irrigations were performed at 0.15 m depth (at 16 and 33 days after transplanting; **Figures 1A,D**) in HW treatment. In IW condition, plants faced a slow reduction of Ψ_m_ and there was no irrigation, with the lowest Ψ_m_ values (between -65 and -90 kPa, depending on genotype) being reached at 0.15 m depth at the end of the experimental period. In order to simulate very harsh conditions, Ψ_m_ reached values close to -150 kPa before irrigations in LW treatment. Irrigations were done at 16, 23, and 29 days after transplantation (**Figure 1**). According to Inman-Bamber et al. (1998) and Silverio et al. (2017), Ψ_m_ between -60 and -100 kPa can be considered a condition of severe water stress for sugarcane at the initial developmental stages. Ψ_m_ was monitored on daily basis and the study was finished 1 day after the first root has reached the bottom of the rhizotron (0.90 m depth), which happened 35 days after transplanting. Differential water supply at the transplanting was the reason for differential dynamics of Ψ_m_ between IW and LW treatments before irrigations.

### Physiological Traits

Leaf gas exchange and photochemistry were evaluated with an infrared gas analyzer (IRGA) model LI-6400 (LICOR Inc., United States) equipped with a fluorometer (6400-40 LCF, LICOR Inc., United States). Measurements were taken between 10:00 and 12:00 a.m., under *Q* of 2,000 μmol m^-2^ s^-1^ and air CO_2_ partial pressure (*C*_a_) of 39.2 ± 0.1 Pa. During measurements, leaf-to-air vapor pressure difference (VPDL) and air temperature inside the IRGA’s cuvette were 3.0 ± 0.9 kPa and 28.9 ± 3.8°C, respectively. We evaluated the leaf CO_2_ assimilation (*A*_N_), stomatal conductance (*g*_S_), transpiration (*E*), intercellular CO_2_ concentration (*C*_I_), intrinsic (WUE_i_ = *A*_N_/*g*_S_) and actual (WUE = *A*_N_/*E*) instantaneous photosynthetic water use efficiency and instantaneous carboxylation efficiency (*k* = *A*_N_/*C*_I_) at 22, 24, 26, 29, and 35 days after transplanting, when plants had fully expanded leaves. Simultaneously to leaf gas exchange, chlorophyll fluorescence emission was evaluated and some photochemical variables were estimated according to Edwards and Baker (1993), Bilger et al. (1995), and Baker (2008): the operating efficiency of photosystem II (*F*_q_′/*F*_m_′); the photochemical quenching (*F*_q_′/*F*_v_′); and the apparent electron transport rate (ETR = *F*_q_′/*F*_m_^′∗^*Q*^∗^0.87^∗^0.4, in which 0.4 is the fraction of light distributed to PSII and 0.87 is the fraction of light absorbed by leaves). The potential quantum efficiency of photosystem II (*F*_v_/*F*_m_) in dark-adapted leaves (30 min), the non-photochemical quenching (NPQ) and the relative excess of light energy (*Q*_EXC_) were evaluated 35 days after transplanting. At this time the CO_2_ fixation efficiency (ϕCO_2_) was also calculated as ϕCO_2_ = *A*_N_/(*Q*^∗^α), where α means the light absorption by the leaf (0.87).

After 35 days of treatment, leaves were collected around 10:00 a.m. and dried in an oven with forced air circulation at 60°C (MA032, Marconi, Brazil) for quantifying carbohydrate concentration. The dry samples were ground and soluble sugars extracted with a solution of methanol:chloroform:water (15:5:3 v/v) for 72 h (Bielesk and Turner, 1966). The supernatant was used to determine the concentration of sucrose (Suc) according to Van Handel (1968). Starch (St) concentration was determined through the enzymatic method proposed by Amaral et al. (2007). Based on leaf dry mass (LDM) of each plant, we estimated the total amount of starch and sucrose stored in plant shoots of both varieties.

### Morphological Traits

Plant height (*H*), assessed from the soil surface until the insertion of leaf +1 (first leaf with visible ligule), was monitored with a regular rule during the experimental period. After 35 days of treatment, plant shoots were collected and the soil was carefully removed from rhizotrons to keep roots intact. Afterward, shoots and roots were separated. The LA was determined with a planimeter (LI-3000C, LICOR Inc., United States) and leaves and shoots were dried at 60°C for obtaining LDM and shoot dry mass (SDM), respectively. Specific leaf area (SLA) was calculated as the ratio between the LA and LDM.

Roots were gently stretched aside a measuring tape to determine the maximum length and then separated into three layers: 0.0–0.30 m (top position), 0.31–0.60 m (intermediate position), and 0.61–0.90 m depth (bottom position). A sub-sample measuring 0.08 m length was collected at the middle portion of each layer and used in image analysis. Roots were stored in ethanol solution (30% v/v) at 4°C until the image analyses were performed. The samples were carefully separated and arranged in a scanner (Scanjet G2410, Hewlett-Packard, United States), avoiding root overlapping. After digitalization, images were analyzed with Safira^®^ software (Stonway, Brazil) in order to estimate root length (RL), root area (RA), root volume (RV) and root diameter (RD). Then, root dry mass (RDM) of each root fraction was determined and RL, RA and RV estimated on root biomass basis. Total dry mass (TDM) was calculated as the sum of SDM and RDM, specific root length (SRL) was obtained as RL/RDM and specific root area (SRA) as RA/RDM (Meier and Leuschner, 2008). The ratio between root and SDM (R/S) was also estimated.

### Phenotypic Plasticity: Changes in Morphological and Physiological Traits

The relative distance plasticity index (RDPI) was calculated following Valladares et al. (2006). The data obtained at 35 days after transplanting were used to calculate RDPI, which indicates the relative phenotypic distance between individuals of the same genotype exposed to different environments, ranging from 0 (no plasticity) to 1 (maximum plasticity). For each sugarcane variety, we built a matrix (3 × 3) of each physiological and morphological parameter, where the rows (*i*) represented the water availability treatments and the columns (*j*) mean the sugarcane individuals, i.e., the replicate under each water treatment. We considered three water regimes (*i* = 1, 2, 3) and three individuals of each sugarcane variety (*j* = 1, 2, 3). The phenotypic plasticity for a given variable *x* can be related to the difference of *x* between two individuals of the same variety grown under different water availability. So, phenotypic plasticity was described by the absolute distance between two selected individuals (*j* and *j*′) of the same variety grown under distinct environments (*i* and *i*′). Regarding this assumption to whole data set, we computed pairwise distances across all individuals and environments. For a given variable *x*, the distance among values (*dij*→*i*′*j*′) is the difference *xi*′*j*′ - *xij* and the relative distances (*rdij*→*i*′*j*′) are defined as *dij*→*i*′*j*′/(*xi*′*j*′ + *xij*) for all pairs of individuals of a given variety grown under different water availability. Finally, RDPI was calculated as Σ(*rdij*→*i*′*j*′)/*n*, where *n* means the number of distances. Detailed description of RDPI and its bases are giving in Valladares et al. (2006). We calculated RDPI considering only physiological traits (RDPI_P_) or the morphological ones (RDPI_M_) as the overall average of RDPI for each physiological (*A*_N_, *g*_S_, *C*_I_, *E*, ϕCO_2_, WUE, WUE_i_, *k*, *F*_q_′/*F*_m_′, *F*_q_′*/F*_v_′, ETR, *F*_v_/*F*_m_, NPQ, and *Q*_EXC_) or morphological (RA, RV, RL, RDM, RD, SDM, LA, SLA, R/S, *H*, TDM, SRL, and SRA) trait, respectively.

### Data Analysis

The experimental design was completely randomized in a 2 × 3 factorial scheme, with two causes of variation given by sugarcane varieties and water availability, and three replicates (one plant per plot). Leaf gas exchange and photochemical parameters were evaluated five times after transplanting, but the statistical analysis revealed no differences among evaluation times. Therefore, average values of 15 observations (three replicates × five times) were used to evaluate the effects of water regimes on leaf gas exchange and photochemistry. The software Assistat 7.6 Beta^®^ (UFPB, Brazil) was used in statistical analyses, being data normality checked before the analysis of variance. When significant differences were detected, mean values of physiological and morphological traits were compared by the Student’s *t*-test (*P* < 0.05). ANOVA results are shown in Supplementary Table 1.

The RDPI was compared by the non-parametric Mann–Whitney test (*P* < 0.05) and all physiological parameters were used for RDPI_P_ estimation, whereas all morphological parameters were considered for RDPI_M_.

## Results

### Soil Water Availability

During the experimental period, water availability was monitored and controlled independently along the soil profile. Ψ_m_ was maintained higher than -20 kPa in the entire soil profile under HW condition (**Figure 1**). Plants faced Ψ_m_ lower than -20 kPa (at 0.15 m depth) from the 18th day under IW condition (**Figures 1A–D**). Under LW condition, Ψ_m_ was lower than -50 kPa since the 8th day of treatment and reached -200 kPa at 0.15 m depth (**Figures 1A–D**). In such treatment, Ψ_m_ reached very low values even at 0.45 and 0.75 m depths (**Figures 1B,C,E,F**). Differences in Ψ_m_ induced by sugarcane genotypes were noticed under IW condition, with IACSP95-5000 causing lower Ψ_m_ than IACSP94-2094 from the 28th day of treatment (**Figure 1**). Under LW condition, IACSP95-5000 reduced Ψ_m_ more than IACSP94-2094 at 0.15 and 0.45 m depths (**Figures 1A,B,D,E**). In such water conditions, the Ψ_m_ variation at 0.75 m depth (**Figures 1C,F**) was due to natural water movement from the wet bulb to the dryer soil and not due to water extraction by plants. In fact, we did not find roots below 0.5 m depth for both sugarcane varieties 15 days after beginning the experimental period.

### Gas Exchange and Photochemistry under Water Deficit

**FIGURE 2:**
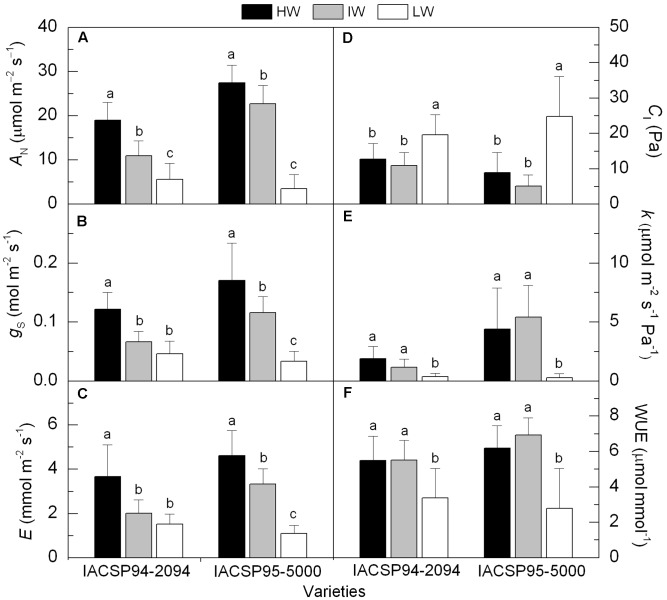
CO_2_ assimilation (*A*_N_, in **A**), stomatal conductance (*g*_S_, in **B**), transpiration (*E*, in **C**), intercellular CO_2_ partial pressure (*C*_I_, in **D**), instantaneous carboxylation efficiency (*k*, in **E**) and actual photosynthetic water use efficiency (WUE, in **F**) of sugarcane varieties IACSP94-2094 and IACSP95-5000 exposed to high (HW, in black), intermediate (IW, light gray) and low (LW, white) water availability. The histograms represent mean values (*n* = 15) + SD. Lowercase letters compare water regimes in each variety (Student’s *t*, *p* < 0.05).

The sugarcane plants subjected to IW and LW conditions showed significant reduction in leaf CO_2_ assimilation (*A*_N_) when compared to plants under HW availability (**Figure 2A**). The impairment of *A*_N_ was highest under LW conditions for both genotypes, but IACSP95-5000 showed the highest relative reduction (-87%). Comparing the varieties, we noticed that IACSP95-5000 presented higher (*p* < 0.05) *A*_N_ than IACSP94-2094 under well-watered and IW conditions (**Figure 2A**). Stomatal conductance (*g*_S_) was also reduced due to water deficit. While IACSP94-2094 showed similar *g*_S_ under IW and LW conditions (**Figure 2B**), a distinct pattern was found in IACSP95-5000. This latter presented the lowest *g*_S_ under LW condition, with reductions of 81% in relation to the well-watered plants. The leaf transpiration (*E*) followed the same pattern of *g*_S_ (**Figures 2B,C**). The intercellular CO_2_ partial pressure (*C*_I_) was increased under LW in both varieties (**Figure 2D**). Significant (*p* < 0.05) reduction in the instantaneous carboxylation efficiency (*k*) was noticed under LW treatment in both varieties, with IACSP95-5000 being more sensitive than IACSP94-2094 (**Figure 2E**). The actual photosynthetic water use efficiency (WUE) was reduced in IACSP94-2094 and IACSP95-5000 under LW condition (**Figure 2F**). Similar response was found for the intrinsic photosynthetic water use efficiency (WUE_i_), with this trait being reduced from 164.9 to 109.3 μmol mol^-1^ in IACSP94-2094 and from 198.2 to 62.9 μmol mol^-1^ in IACSP95-5000 when comparing HW and LW treatments.

**FIGURE 3:**
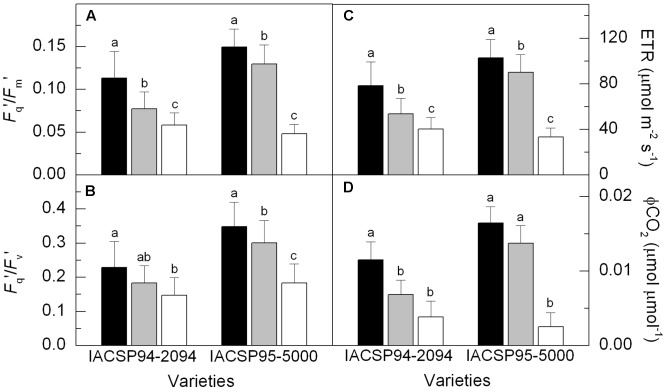
Operating efficiency of photosystem II (*F*_q_′/*F*_m_′, in **A**), photochemical quenching (*F*_q_′/*F*_v_′, in **B**), apparent electron transport rate (ETR, in **C**) and CO_2_ fixation efficiency (ϕCO_2_, in **D**) of sugarcane varieties IACSP94-2094 and IACSP95-5000 exposed to high (HW, black), intermediate (IW, light gray) and low (LW, white) water availability. The histograms represent mean (*n* = 15) + SD. Lowercase letters compare water regimes in each variety (Student’s *t*, *p* < 0.05).

Significant reduction in the potential quantum efficiency of photosystem II (*F*_v_/*F*_m_) was found only in IACSP95-5000 under LW condition (Supplementary Figure 1A). However, primary photochemistry in light-adapted leaves was reduced by water deficit in both varieties (**Figure 3**), with IACSP95-5000 being more sensitive than IACSP94-2094 when considering the operating efficiency of photosystem II (*F*_q_′*/F*_m_′), the photochemical quenching (*F*_q_′/*F*_v_′), the apparent ETR and CO_2_ fixation efficiency (ϕCO_2_). The low water availability increased the relative excess of light energy (*Q*_EXC_) at PSII level in both varieties (Supplementary Figure 1B). However, IACSP95-5000 presented higher sensitivity of *Q*_EXC_ to water withholding. Water deficit did not affect NPQ, with IACSP94-2094 and IACSP95-5000 showing average values of 1.35 ± 0.66 and 1.44 ± 0.70, respectively.

### Plant Growth under Water Deficit

**FIGURE 4:**
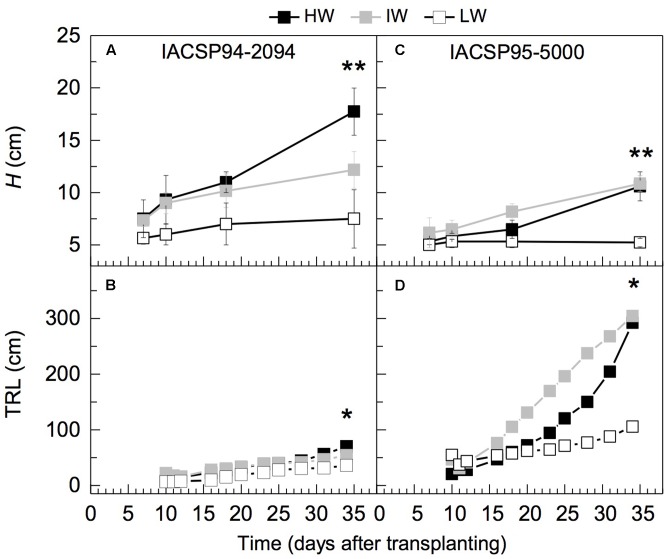
Temporal variation of plant height (*H*, in **A,C**) and visible root length (TRL, in **B,D**) on rhizotrons glass window of two sugarcane varieties IACSP94-2094 **(A,B)** and IACSP95-5000 **(C,D)** exposed to high (HW, in black), intermediate (IW, light gray) and low (LW, white) water availability. Each symbol represent means (*n* = 3) ± SD. ^∗,∗∗^ mean statistical difference at *p* < 0.05 and *p* < 0.01, respectively.

After 35 days of experiment, plant height of IACSP94-2094 was reduced under IW and LW conditions, whereas only LW caused reduction of this trait in IACSP95-5000 (**Figures 4A,C**). The total visible length of roots revealed that IACSP94-2094 has slow root growth regardless water regime, whereas IACSP95-5000 showed fast root growth, mainly under IW condition (**Figures 4B,D**). In general, both varieties presented reduction of RL under LW condition.

Both IACSP94-2094 and IACSP95-5000 presented decreases in SDM and RDM under LW condition (**Table 1**). However, IACSP95-5000 showed an important increase in RDM under IW treatment (+47%). This pattern was not found in IACSP94-2094 as SDM and RDM were quite similar among HW and IW treatments. When comparing varieties, IACSP95-5000 showed higher biomass than IACSP94-2094, regardless the water regime. In general, RDM, RA and RL presented the same pattern of response when considering water regimes and genotypes. Regarding the biomass partitioning between roots and shoots (R/S), IACSP94-2094 presented a significant reduction under LW whereas IACSP95-5000 exhibited a large increase (1.8 times) under IW condition. The RD and SRA of both varieties were not affected by water regimes. The RV was reduced in IACSP94-2094 and IACSP95-5000 under LW condition; however, we noticed a large increase of RV in IACSP95-5000 under IW condition. The SRL of IACSP94-2094 was significantly increased under IW and decreased under LW. On the other hand, SRL of IACSP95-5000 was not changed due to water regimes. There were reductions in LA (-34%) and SLA (-23%) of IACSP94-2094 under IW, with such changes being intensified under LW condition. Both LA and SLA of IACSP95-5000 were reduced only under LW treatment (**Table 1**).

**Table 1:** Shoot dry mass (SDM), root dry mass (RDM), root area (RA), root length (RL), ratio between root and shoot dry mass (R/S), mean root diameter (RD), specific root area (SRA), root volume (RV), specific root length (SRL), leaf area (LA) and specific leaf area (SLA) of sugarcane varieties IACSP94-2094 and IACSP95-5000 grown under high (HW), intermediate (IW) and low (LW) water availability.

Variety	WR	SDM (g)	RDM (g)	RA (m^2^)	RL (m)	R/S –	RD (mm)	SRA (m^2^ g^-1^)	RV (cm^3^)	SRL (m g^-1^)	LA (cm^2^)	SLA (cm^2^ g^-1^)
IACSP	HW	2.1 ± 0.4^bA^	0.54 ± 0.05^bA^	0.11 ± 0.02^aA^	75 ± 10^aA^	0.27 ± 0.04^aA^	0.39 ± 0.03^ns^	0.21 ± 0.03^ns^	15 ± 6^bA^	140 ± 7^aA^	125 ± 21^bA^	126 ± 6^aA^
94-2094	IW	1.8 ± 0.4^bA^	0.40 ± 0.11^bA^	0.09 ± 0.04^bA^	52 ± 11^bB^	0.24 ± 0.12^bA^	0.39 ± 0.03	0.21 ± 0.05	18 ± 3^bA^	132 ± 13^aA^	83 ± 20^bB^	97 ± 9^bB^
	LW	0.6 ± 0.3^bB^	0.10 ± 0.07^bB^	0.02 ± 0.02^bB^	10 ± 11^bC^	0.07 ± 0.08^bB^	0.47 ± 0.08	0.15 ± 0.07	3 ± 2^aB^	81 ± 47^aB^	10 ± 3^bC^	59 ± 14^bC^
IACSP	HW	2.8 ± 0.1^aA^	0.83 ± 0.30^aB^	0.13 ± 0.03^aB^	72 ± 12^aB^	0.29 ± 0.10^aB^	0.39 ± 0.02	0.16 ± 0.03	26 ± 9^aB^	91 ± 20^bA^	198 ± 14^aA^	124 ± 5^aA^
95-5000	IW	2.9 ± 0.4^aA^	1.56 ± 0.17^aA^	0.25 ± 0.02^aA^	139 ± 14^aA^	0.54 ± 0.04^aA^	0.43 ± 0.05	0.16 ± 0.01	45 ± 3^aA^	90 ± 9^bA^	187 ± 10^aA^	122 ± 8^aA^
	LW	1.1 ± 0.3^aB^	0.31 ± 0.09^aC^	0.06 ± 0.001^aC^	36 ± 7^aC^	0.28 ± 0.02^aB^	0.49 ± 0.15	0.19 ± 0.03	10 ± 1^aC^	118 ± 9^aA^	64 ± 8^aB^	99 ± 13^aB^

*Distinct lowercase letters indicate statistical difference (Student’s t-test, p < 0.05) between varieties in the same water regime (WR), whereas capital letters differentiate water regimes in a given variety. ^ns^indicates non-significant difference. Mean values (n = 3) ± SD.*

**FIGURE 5:**
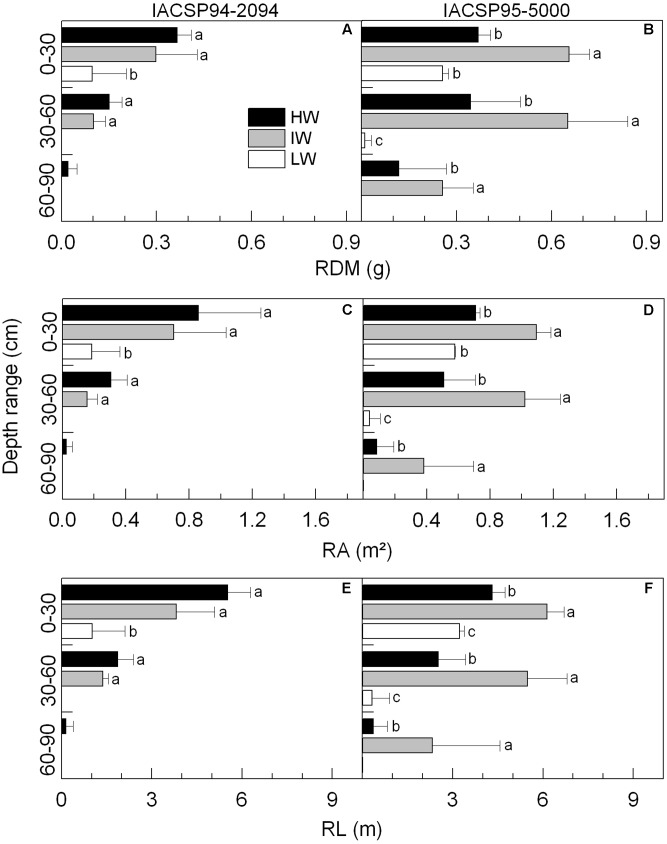
Soil profiles of root dry mass (RDM, in **A,B**), root area (RA, in **C,D**) and root length (RL, in **E,F**) in sugarcane varieties IACSP94-2094 (in **A,C,E**) and IACSP95-5000 (in **B,D,F**) exposed to high (HW, black), intermediate (IW, light gray), and low (LW, white) water availability. Bars indicate the mean value (*n* = 3) + SD. Lowercase letters compare water regimes (Student’s *t*, *p* < 0.05) in a given variety and soil depth.

Along the soil profile, IACSP95-5000 increased the amount of roots and no changes were found in IACSP94-2094 under IW availability, as compared to HW condition (**Figures 5A,B**). Profiles of RA and RL presented the same patterns of variation due to water regimes, with both varieties showing reductions under LW and only IACSP95-5000 showing increases in RA and RL under IW condition (**Figures 5C–F**).

### Leaf Carbohydrate Content under Water Deficit

**FIGURE 6:**
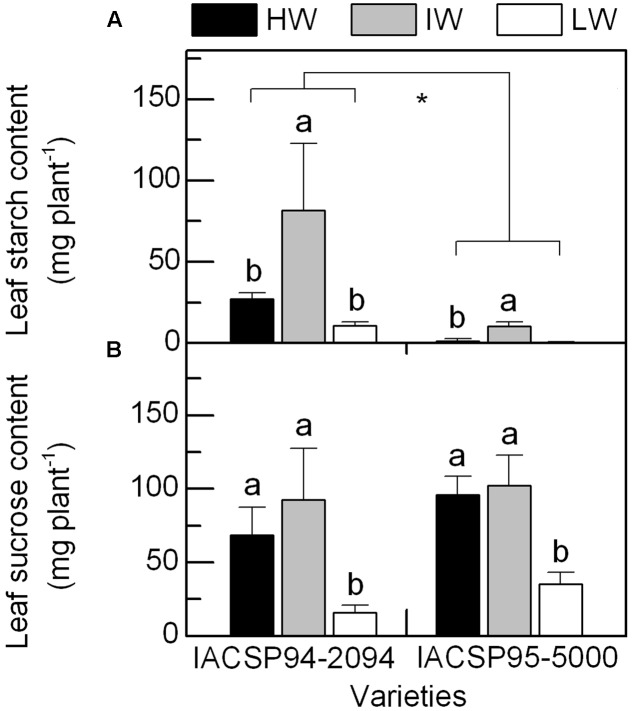
Total content of starch (in **A**) and sucrose (in **B**) in leaves of sugarcane varieties IACSP94-2094 and IACSP95-5000 exposed to high (HW, black), intermediate (IW, light gray) and low (LW, white) water availability. Each histogram represents the mean (*n* = 3) + SD. Lowercase letters compare water regimes for a given variety, while asterisk indicates statistical differences between varieties (Student’s *t*, *p* < 0.05).

As compared to the well-watered condition, IACSP94-2094 and IACSP95-5000 presented increases in leaf starch content under IW condition (**Figure 6A**), without significant changes in sucrose content (**Figure 6B**). When comparing HW and LW treatments, we noticed decreases in sucrose content and no changes in starch content in both varieties due to water deficit. We were not able to detect starch in leaves of IACSP95-5000 under LW condition. Although similar responses have been found in both varieties, IACSP95-5000 presented leaf starch content much lower than IACSP94-2094 in all water regimes (**Figure 6A**). The ratio sucrose/starch varied from 1.1 (IW) to 2.5 (HW) in IACSP94-2094 and from 9.7 (IW) to 85.6 (LW) in IACSP95-5000 across the water regimes.

### Phenotypic Plasticity

**FIGURE 7:**
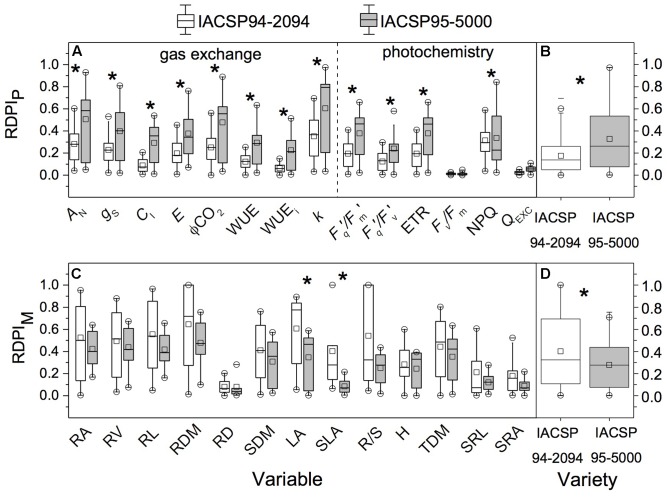
Individual (for each trait, in **A,C**) and overall (average of all traits, in **B,D**) relative distance plasticity index considering physiological (RDPI_P_) and morphological (RDPI_M_) traits in sugarcane varieties IACSP94-2094 and IACSP95-5000 subjected to varying water availability. ^∗^means significant difference between varieties (*p* < 0.05, Mann–Whitney’s test). *A*_N_, leaf CO_2_ assimilation; *g*_S_, stomatal conductance; *C*_I_, intercellular CO_2_ partial pressure; *E*, transpiration; ϕCO_2_, CO_2_ fixation efficiency; WUE, actual photosynthetic water use efficiency; WUE_i_, intrinsic photosynthetic water use efficiency; *k*, instantaneous carboxylation efficiency; *F*_q_′/*F*_m_′, operating efficiency of photosystem II; *F*_q_′/*F*_v_′, photochemical quenching; ETR, apparent electron transport rate; *F*_v_/*F*_m_, potential quantum efficiency of photosystem II; NPQ, non-photochemical quenching; *Q*_EXC_, relative excess of light energy; RA, root area; RV, root volume; RL, root length; RDM, root dry mass; RD, root diameter; SDM, shoot dry mass; LA, leaf area; SLA, specific leaf area; R/S, root:shoot ratio; *H*, height; TDM, total dry mass; SRL, specific root length; SRA, specific root area.

IACSP95-5000 showed higher RDPI of leaf gas exchange (*A*_N_, *g*_S_, *C*_I_, *E*, ϕCO_2_, WUE, WUE_i_ and *k*) and photochemistry (*F*_q_′/*F*_m_′, *F*_q_′/*F*_v_′, ETR, and NPQ) than IACSP94-2094 (**Figure 7A**). Taking into account the morphological traits, IACSP94-2094 showed higher plasticity of LA and SLA than IACSP95-5000 (**Figure 7C**). In general, IACSP95-5000 presented higher RDPI_P_ than IACSP94-2094 (**Figure 7B**) while this latter had higher RDPI_M_ than IACSP95-5000 (**Figure 7D**).

## Discussion

### Morphological and Physiological Sensitivity of Sugarcane to Water Deficit

Although there were no data about leaf water potential, we may argue that plants under IW and LW conditions were experiencing water deficit once physiology and/or growth of those plants were affected (**Figures 2**–**5**). Taking into account plant responses (**Figures 2**–**5**) and also soil water availability (**Figure 1**), we may consider that IW represents a slight water deficit whereas LW represents a severe water deficit. In general, the physiological responses to IW and LW treatments reported herein are already known and described in recent papers (Machado et al., 2009, 2013; Ribeiro et al., 2013; Sales et al., 2013). For instance, reductions in stomatal conductance, carboxylation and photochemistry were associated with the inhibition of sugarcane photosynthesis (**Figures 2**, **3**) and such responses were genotype-dependent. However, this study shows that conclusions on sugarcane sensitivity to drought are affected by the morphological and physiological variables considered and also by the intensity of water deficit.

The physiological traits revealed that IACSP94-2094 was more sensitive to slight water deficit (IW treatment) than IACSP95-5000 (**Figures 2**, **3**). Considering the morphological traits, IACSP95-5000 had improved performance under IW as compared to HW, accumulating more biomass due to increases in root growth (**Table 1** and **Figure 5**). Under IW condition, IACSP95-5000 showed higher water extraction than IACSP94-2094 in all three soil depths (**Figure 1**). Then, we may argue that increases in root growth improved soil volume exploration, indicating an important morphological response under marginally reduced water availability. In general, sugarcane performance under water deficit has been evaluated considering only shoot responses (Inman-Bamber and Smith, 2005; Basnayake et al., 2012; Machado et al., 2013; Ribeiro et al., 2013; Santos et al., 2015), even knowing that plants should maximize the uptake of water and nutrient use to tolerate water deficit (Blum, 2005). Our data revealed that IACSP95-5000 presents higher ability than IACSP94-2094 in acquiring soil water at the initial growth stage when plants face reductions in water availability. From a practical point of view, this finding suggests that IACSP95-5000 may overcome slight water deficit in new sugarcane fields, where limiting conditions may occur just after sprouting or during the initial growth.

As compared to IACSP94-2094, the lower height and higher biomass of IACSP95-5000 under well-watered conditions suggest that stalks were thicker in this latter. Interestingly, IACSP95-5000 showed higher biomass production than IACSP94-2094 under HW and IW conditions and this may be explained by its higher photosynthetic performance (**Figure 2A**). In fact, photosynthate supply is critical for root growth as it depends on oxidation of carbohydrates to produce energy and carbon skeletons. At this time, we may suggest that IACSP95-5000 could transport more efficiently photosynthate to roots, which is supported by low leaf starch content even with plants showing high photosynthetic rates under HW condition (**Figures 2A**, **6A**). Due to improved root growth, IACSP95-5000 produced more biomass under IW as compared to HW and this fact cannot be explained by changes in leaf or canopy photosynthesis (**Figure 2A** and **Table 1**). The other component of plant carbon balance is respiration and we know that the respiration efficiency is increased in maize roots under stressful conditions (Machado and Pereira, 1990). As IACSP95-5000 showed higher biomass conversion efficiency under IW condition, our data indicate that plant carbon balance under drought conditions is dependent on sugarcane genotype, and root respiration may play an important role in sugarcane performance under water deficit (Flexas et al., 2006).

Considering the relative difference in dry mass production between plants under HW and LW conditions (**Table 1**), IACSP95-5000 and IACSP94-2094 had similar sensitivity to water deficit. However, the physiological data indicated that IACSP95-5000 was more sensitive to LW condition when compared to IACSP94-2094. Such higher sensitivity of IACSP95-5000 to water deficit was associated with a large impairment in photosynthetic metabolism under LW, with plants showing significant reductions in stomatal conductance (**Figure 2B**), carboxylation (**Figure 2E**) and primary photochemistry (**Figure 3A**). Clearly, IACSP94-2094 presents physiological mechanisms to limit the drought-induced damage on photosynthesis that are activated after slight reductions in soil water availability and prevents further decreases of photosynthesis under moderate to severe water deficit.

### Phenotypic Plasticity of Sugarcane under Water Deficit

When studying sugarcane responses to water deficit, several morphological and physiological traits have been evaluated and reported (Machado et al., 2009, 2013; Ribeiro et al., 2013; Sales et al., 2013). Such large dataset of numbers and variables turns difficult an overall idea of how water deficit affects plants and how plants respond to such limiting condition. As an attempt to have an integrative index related to plant sensitivity to drought, we estimated the phenotypic plasticity of two sugarcane varieties under reducing water availability. In fact, phenotypic plasticity has been used to study plant acclimation to environmental changes and to understand the growth capacity under limiting conditions (Valladares et al., 2006). Although both genotypes had presented relatively low plasticity (RDPI values lower than 0.5), IACSP95-5000 showed higher physiological plasticity than IACSP94-2094 (**Figures 7A,B**), which was based on all photosynthetic parameters evaluated with exception of *F*_v_/*F*_m_ and *Q*_EXC_. As IACSP95-5000 presented higher biomass than IACSP94-2094 under LW treatment (**Table 1**), we may argue that physiological plasticity benefits plant growth under water limiting conditions. Accordingly, the physiological plasticity is an important plant trait as physiological responses generally occur in short-term, are reversible and the energy costs involved are lower when compared to morphological changes (Meier and Leuschner, 2008).

Regarding the morphological plasticity, IACSP94-2094 presented higher plasticity than IACSP95-5000 and this pattern was based on variations in LA and SLA (**Figures 7C,D**). A large reduction in LA is a well-known strategy to reduce plant transpiration and water consumption with negative side-effect on canopy photosynthesis. However, the effectiveness of this morphological strategy for maintaining leaf water status was not proven herein as both varieties presented similar stomatal conductance under LW condition (**Figure 2B**). On the other hand, decreases in SLA suggest that leaves became thicker and proteins more concentrated on area basis. This latter would be a strategy of IACSP94-2094 to reduce the negative impact of water deficit on photosynthesis as a large fraction of leaf proteins is involved in the photosynthetic apparatus (Makino et al., 2003). In fact, the photosynthesis of IACSP94-2094 was less affected by LW treatment as compared to IACSP95-5000 (**Figure 2**) and such photosynthetic tolerance of IACSP94-2094 to water deficit was already reported by Ribeiro et al. (2013). In general, our data indicate that morphological plasticity does not always represent an effective advantage for maintaining plant growth under water deficit. When the effectiveness of physiological and morphological changes in maintaining plant growth under low water availability is taking into account (**Figure 7** and **Table 1**), our data indicate that the physiological plasticity was more important than the morphological one in young sugarcane plants under water deficit. We were able to recognize different strategies of sugarcane under low water availability, with IACSP95-5000 presenting higher physiological plasticity and IACSP94-2094 showing higher morphological plasticity. As this study was carried out with young plants, further research is needed to evaluate if morphological and physiological responses found herein are sustained under field conditions.

## Author Contributions

Conception and design of this study (PM, EM, RP, and RR). Collection of data (PM, CS, EE-N, and JMF). Analysis and interpretation of the data (PM, EM, GS, RP, and RR). Drafting of the article (PM and RR). Critical revision and final approval of the article (all authors).

## Conflict of Interest Statement

The authors declare that the research was conducted in the absence of any commercial or financial relationships that could be construed as a potential conflict of interest.
