# Supplementary material for: Physiological Plasticity Is Important for Maintaining Sugarcane Growth under Water Deficit
Source: Front Plant Sci. 2017 Dec 20;8:2148. doi: 10.3389/fpls.2017.02148 (PMC5742411; doi:10.3389/fpls.2017.02148)
Supplement: Supplementary file 1 [file Table_1.DOCX]

Supplementary Material

Physiological Plasticity Is Important For Maintaining Sugarcane Growth Under Water Deficit

**Paulo E. R. Marchiori, Eduardo C. Machado, Cristina R. G. Sales, Erick Espinoza-Núñez, José R. Magalhães-Filho, Gustavo M. Souza, Regina C. M. Pires and Rafael V. Ribeiro***

*** Correspondence:** Corresponding Author: rvr@unicamp.br

# Supplementary Figures and Tables

## Supplementary Figure

**
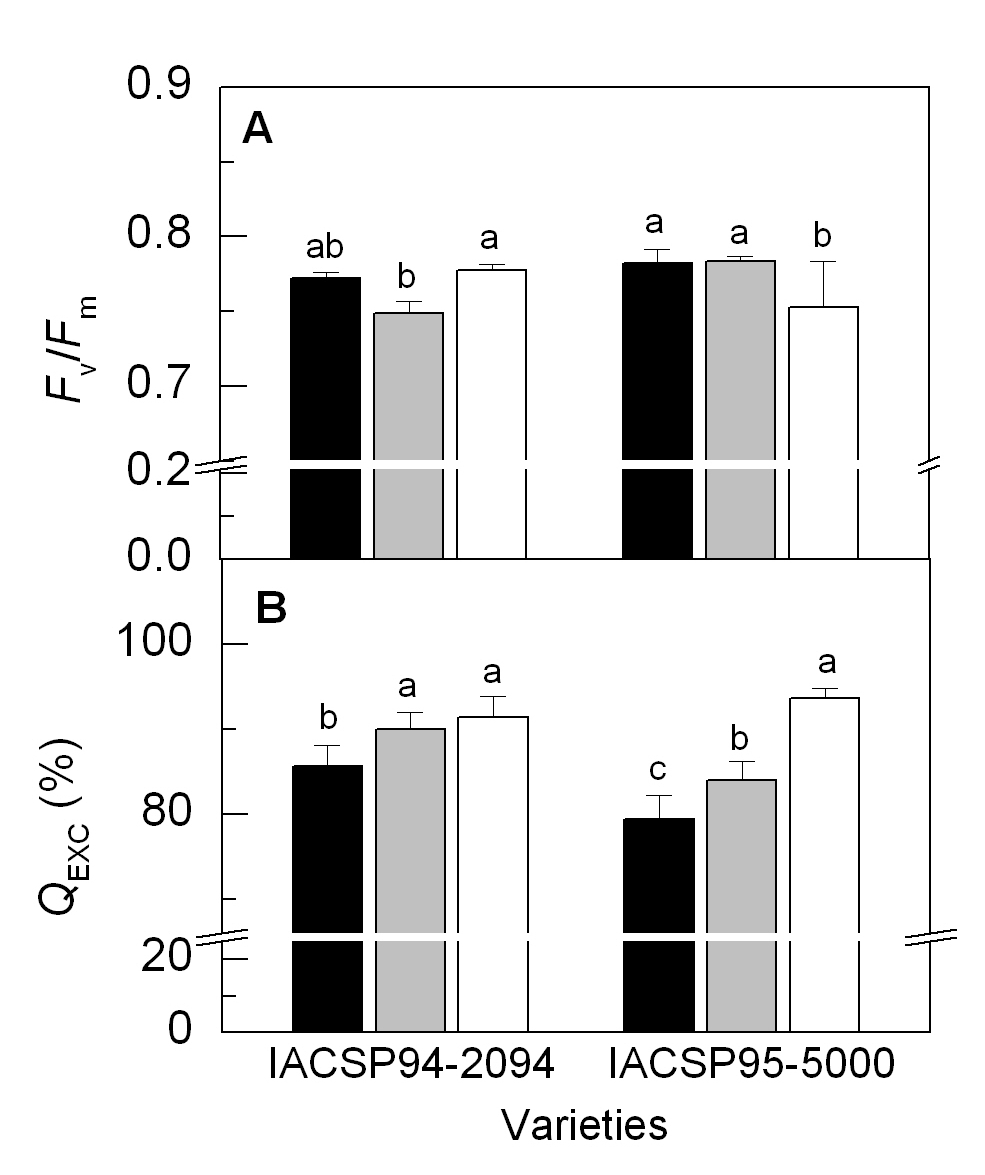
**

**Supplementary Figure 1.** Potential quantum efficiency of photosystem II (*F*_v_/*F*_m_, in A) and relative excess of light energy (*Q*_EXC_, in B) of sugarcane varieties IACSP94-2094 and IACSP95-5000 exposed to high (HW, black), intermediate (IW, light gray) and low (LW, white) water availability. The histograms represent mean (n=3) + s.d. Lowercase letters compare water regimes in each variety (Student's t, *p*<0.05).

## Supplementary Table

**Supplementary Table 1.** Results from analysis of variance (degree of freedom, *DF*; means square, *MS*) for physiological and morphological variables considering two sugarcane varieties (Var.) and three water regimes (WR) as sources of variation, and their interaction. **,* means significant at *p*<0.01 and *p*<0.05, respectively.

| Source of  variation | DF | Variables**^§^** | | | | | | | | | | | | | |
| --- | --- | --- | --- | --- | --- | --- | --- | --- | --- | --- | --- | --- | --- | --- | --- |
|  |  | *A*_N_ | *g*_S_ | *C*_I_ | *E* | *Φ*CO_2_ | WUE | WUE_i_ | *k* | *F*_q_’/*F*_m_’ | *F*_q_’/*F*_v_’ | ETR | *F*_v_/*F*_m_ | NPQ | *Q*_EXC_ |
| Var. | 1 | 205.6^**^ | 0.005^**^ | 19.08^ns^ | 1.91^**^ | 0.00008^**^ | 2.70^ns^ | 598.0^ns^ | 4.69^**^ | 0.0032^**^ | 0.017^**^ | 1567^**^ | 0.0002^ns^ | 0.033^ns^ | 0.005^**^ |
| WR | 2 | 338.8^**^ | 0.010^**^ | 123.12^**^ | 6.71^**^ | 0.00011^**^ | 10.89^**^ | 4325.8^**^ | 4.06^**^ | 0.0093^**^ | 0.023^**^ | 4546^**^ | 0.0003^ns^ | 0.330^ns^ | 0.018^**^ |
| Var. x WR | 2 | 113.1^*^ | 0.003^*^ | 57.69^**^ | 1.57^**^ | 0.00004^**^ | 4.54^**^ | 2068.2^**^ | 1.74^**^ | 0.0026^**^ | 0.004^ns^ | 1212^**^ | 0.0014^**^ | 0.210^ns^ | 0.002^*^ |

*Continue….*

| Source of  variation | DF | Variables**^§^** | | | | | | | | | | | | | | |
| --- | --- | --- | --- | --- | --- | --- | --- | --- | --- | --- | --- | --- | --- | --- | --- | --- |
|  |  | RA | RV | RL | RDM | RD | SDM | LA | SLA | R/S | *H* | TDM | SRL | SRA | St. | Suc. |
| Var. | 1 | 0.025^**^ | 1001^**^ | 6019^**^ | 1.39^**^ | 0.0002^ns^ | 3.09^**^ | 26380^**^ | <0.01^ns^ | 0.14^**^ | 257.3^**^ | 8.92^**^ | 1486^ns^ | 0.015^ns^ | 10039^**^ | 1720^**^ |
| WR. | 2 | 0.016^*^ | 837^**^ | 8358^**^ | 0.92^**^ | 0.007^ns^ | 4.82^**^ | 25744^**^ | <0.01^ns^ | 0.08^**^ | 94.4^**^ | 9.83^**^ | 406^ns^ | 0.002^ns^ | 1540^*^ | 1140^**^ |
| Var. x WR | 2 | 0.005^**^ | 257^**^ | 3235^**^ | 0.42^**^ | 0.007^ns^ | 0.15^ns^ | 965.85^*^ | <0.01^ns^ | 0.03^*^ | 14.66 | 1.00^*^ | 3452^**^ | 0.036^ns^ | 971.7^ns^ | 361.7^ns^ |

**^§^***A*_N_ = leaf CO_2_ assimilation. *g*_S_ = stomatal conductance. *C*_I_ = intercellular CO_2_ concentration. *E* = transpiration. *Φ*CO_2_ = CO_2_ fixation efficiency. WUE = actual photosynthetic water use efficiency. WUE_i_ = intrinsic photosynthetic water use efficiency. *k* = instantaneous carboxylation efficiency. *F*_q_’/*F*_m_’ = operating efficiency of photosystem II. *F*_q_’/*F*_v_’ = photochemical quenching. ETR = apparent electron transport rate. *F*_v_/*F*_m_ = potential quantum efficiency of photosystem II. NPQ = non-photochemical quenching. *Q*_EXC_ = relative excess of light energy. RA = root area. RV = root volume. RL = root length. RDM = root dry matter. RD = root diameter. SDM = shoot dry matter. LA = leaf area. SLA = specific leaf area. R/S = root:shoot ratio. *H* = height. TDM = total dry matter. SRL = specific root length. SRA = specific root area. St = leaf starch. Suc. = leaf sucrose.
